# Effects of Vitamin C on the Oral-Nasal Mucosal Damage Caused by Favipiravir in Old and Young Rats

**DOI:** 10.7759/cureus.28796

**Published:** 2022-09-05

**Authors:** Yücel Kurt, Özlem Özmen

**Affiliations:** 1 Otolaryngology - Head and Neck Surgery, Finike State Hospital, Antalya, TUR; 2 Pathology, Mehmet Akif Ersoy University, Burdur, TUR

**Keywords:** covid-19, pathology, caspase-3, rankl, vitamin c, favipiravir, oral-nasal mucosa

## Abstract

Background

Favipiravir was widely used to treat coronavirus disease 2019 (COVID-19) early in the pandemic. Later, many reports began to be published about the side effects of favipiravir on different tissues. However, the side effects of favipiravir on the oral and nasal mucosa remain unknown. This experimental study aimed to evaluate the pathological effects of favipiravir on the oral-nasal mucosa and investigate whether vitamin C (Vit C) reduces these lesions in old and young rats.

Methodology

A total of 100 rats were used for this study. The rats were administered favipiravir (20 mg/kg and 100 mg/kg) and Vit C (150 mg/kg/day) for 14 days. At the end of the study, rats were euthanatized, and oral-nasal mucosal histopathological changes were evaluated. Nuclear factor kappa-ligand (RANKL) and caspase-3 expressions were immunohistochemically examined.

Results

Favipiravir caused severe lesions in old rats compared to young, and the severity of the lesions increased with the dosage. Severe hyperemia and erosive-ulcerative lesions were observed in the oral-nasal mucosa. In addition, increased RANKL and caspase-3 expressions were observed in a dose-dependent manner. In both young and old groups, Vit C treatment showed decreased caspase-3 and RANKL expression; a more prominent decrease was seen in young rats.

Conclusions

This study showed that favipiravir could cause histopathological lesions in the oral and nasal mucosa. However, the administration of Vit C with favipiravir can provide a protective effect against this damage. The curative effect of Vit C was more pronounced in young rats and at low doses.

## Introduction

The coronavirus disease COVID-19, a severe acute respiratory syndrome that emerged in China in December 2019, quickly became a worldwide pandemic for which humanity was unprepared. COVID-19 infection is a highly contagious disease characterized by coughing, loss of smell or taste, fever, difficulty breathing, nasal congestion, and diarrhea [1-3]. Since the beginning of the pandemic, many drugs have been introduced to treat the disease, but no effective treatment has yet been found. Favipiravir is an antiviral medication, a prodrug (ribofuranosyl-5'-triphosphate) of the purine nucleotide. The active ingredient of favipiravir inhibits RNA polymerase. The drug can inhibit the replication of viruses in the body. Since 2014, favipiravir has been approved for treating cases of influenza resistant to conventional therapy. Because of this attribute, the medication has been used to treat mild-to-moderate cases of COVID-19 [4]. In parallel with the increased use of favipiravir, reports of its side effects have arisen. This drug’s most commonly reported side effects include fever, hyperuricemia, diarrhea, and neutropenia [5]. In experimental animal studies, oral administration of the drug has been shown to cause hepatocytic vacuolization, an increase in liver enzymes, a decrease in erythrocyte production, and alveolar bone loss [6,7]. Favipiravir also has teratogenic effects; therefore, administration of the drug is avoided in pregnant women [8]. However, laboratory and clinical research have shown that favipiravir has fewer side effects than other antiviral drugs when used for treating COVID-19 [5]. Vitamin C (Vit C) is a potent antioxidant that contributes to the protection, improvement, and functioning of various cell types. The most significant pharmacological properties of Vit C, including antioxidant, anti-inflammatory, antiviral, and immunomodulatory effects, have been reported [9]. As in many diseases, Vit C has been shown to have positive results when administered intravenously at high doses for COVID-19 [10]. Vit C and favipiravir are commonly used treatments for COVID-19. Studies on this subject are intensively carried out in many countries [11]. Recent publications suggest that some mucocutaneous lesions may be associated with favipiravir-induced vasculitis [12,13]. A recent experimental study determined that favipiravir causes alveolar bone loss in the jawbone and teeth loss [7]. It is known that side effects of COVID-19 and associated medications are more common in older patients. It is well documented that there are issues with taste and smell over the course of COVID-19 and its treatment. There are theories that suggest both sickness and drug use may contribute to this condition. There is limited data on whether nose and mouth mucous membrane lesions occur after COVID-19 infection and whether these are related to the disease or the drugs used. The present study investigates whether favipiravir has a side effect on the oral and nasal mucosa and the effect of Vit C on these lesions in old and young rats.

## Materials and methods

**Figure 1:**
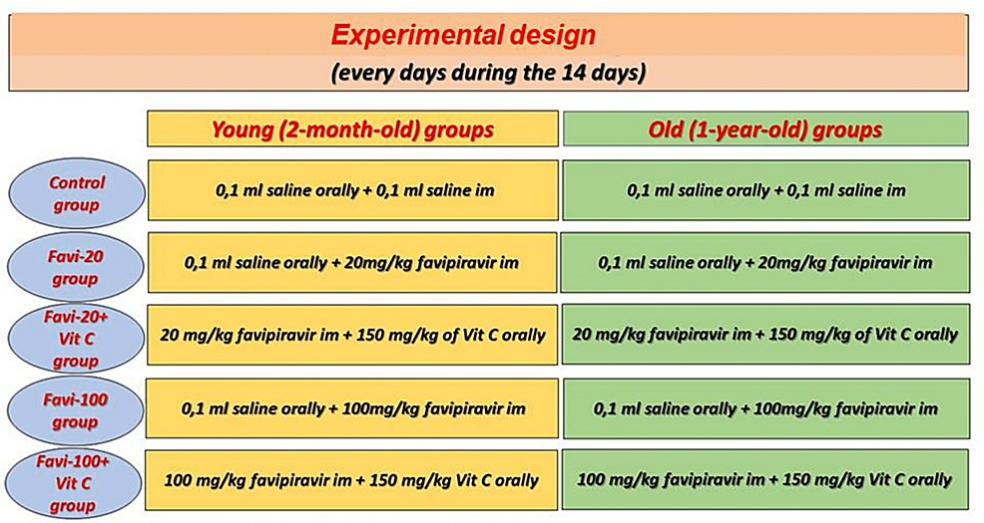
Groups and treatments for groups. Favi: favipiravir; Vit C: vitamin C

All experimental procedures were performed according to the Animal Research: Reporting in Vivo Experiments (ARRIVE) guidelines 2.0. The rats used in the study were kept in controlled rooms with adjusted humidity and temperature throughout the experiment (humidity level 55 ± 5%, 12 hours light/dark cycle, temperature 22 ± 2°C). The rats were given access to ad libitum feed and water until sacrification. A total of 100 healthy, adult male Sprague-Dawley rats (young groups aged two months and old groups aged one year) were used in this study. The rats were randomly divided into 10 equal groups of 10 rats each. According to groups, favipiravir (20 mg/kg and 100 mg/kg, intramuscularly, T705, Merck) and Vit C (150 mg/kg/day, oral, L-ascorbic acid, Merck) were administered to the rats for 14 days. The experimental design is shown in Figure 1.

According to groups, favipiravir (20 mg/kg and 100 mg/kg, intramuscularly (IM), and Vit C (150 mg/kg/day, orally) were administered to the rats for 14 days. The experimental designs are shown in Figure 1. At the end of the experimental procedure, the rats were humanely killed under anesthesia (80-100 mg/kg of ketamine; alfamin, alfasan IBV and 5-7 mg/kg xylazine; alfazin, alfasan IBV). Necropsy head samples were harvested and fixed in a 10% neutral formalin solution.

Histopathological evaluation

After three days of formalin fixation, the head samples were decalcified in 0.1 M ethylenediaminetetraacetic acid (EDTA) solution for two weeks. After the bones were softened, samples were taken from the nasal and oral mucosa and placed in tissue-processing cassettes. Samples were washed under running water for eight hours, and tissues were routinely processed and embedded in paraffin. After two hours of cooling, 5 μm serial sections were taken from the paraffin blocks. To examine different areas of each animal’s oral and nasal mucosa, the samples were divided into three parts and included in the follow-up procedure. Three serial sections were prepared, and two levels of sections were taken from all rats for histopathological and immunohistochemical examinations. One serial section was stained with hematoxylin and eosin (H&E) and examined using a light microscope (Olympus CX41, Olympus Corporation, Tokyo, Japan). Six different fields were examined from mouth and nose samples for a single rat by cross-sectioning from two levels from three samples for each rat.

Immunohistochemical analysis

The other two serial sections were stained to evaluate the expression of caspase-3 (anti-caspase-3 antibody, ab4051) and nuclear factor kappa-ligand (RANKL) (anti-RANKL antibody, ab216484). The streptavidin-biotin peroxidase technique was processed according to the manufacturer’s instructions. All primary antibodies and seconder kits were purchased from Abcam (Cambridge, UK). Both primary antibodies were used at a 1/100 dilution for the analysis. As a secondary kit, an UltraVision Detection System Anti-Polyvalenti HRP kit (TP-060-HL) (Thermo Shandon Limited, Cheshire, UK) and chromogen 3,3'-diaminobenzidine were used. To evaluate the wrong connections we used negative controls, antibody dilution solutions were replaced with the primary antibodies checked under a light microscope to avoid background staining and washed twice. All tissue examinations were performed by a pathologist blinded to the specimen treatments. All slides were analyzed for immunopositivity by semi-quantitative analysis. Samples were analyzed by assessing five different areas in each rat. Immunohistochemical staining was scored between 0 and 3, where 0 indicates negative, 1 mild, 2 moderate, and 3 severe positive [7]. The scores obtained were analyzed statistically and tabulated. An automated image analysis system (Olympus CX41, Olympus Corporation, Tokyo, Japan) was used to obtain computer-assisted histomorphometric measurements and immunohistochemical scoring to support the data after conventional microscopic examination. The Database Manual CellSens Life Science Imaging Software System (Olympus Corporation) was used for histomorphological and immunohistochemical analysis. Immunohistochemical score analyses were performed using Image J 1.46r (National Institutes of Health, Bethesda, MD, USA).

Statistical analysis

Statistical analyses were performed using the SPSS version 21.0 (IBM Corp., Armonk, NY, USA). A one-way analysis of variance test was used to reveal significant differences in immunohistochemical analysis scores between the groups. Duncan test was used to compare the groups, with p-values of <0.05 as the level of significance.

Ethics statement

The animal experiments were approved by the Local Ethics Committee of Experimental Animal Research of Pamukkale University (PAUHDEK-2021/23), Denizli, Turkey.

## Results

None of the rats died during the experiment. Clinically, no significant difference was observed between the groups during the study. All rats in all groups were healthy as confirmed by a responsible veterinarian.

Histopathological findings

Oral Mucosal Findings

**Figure 2:**
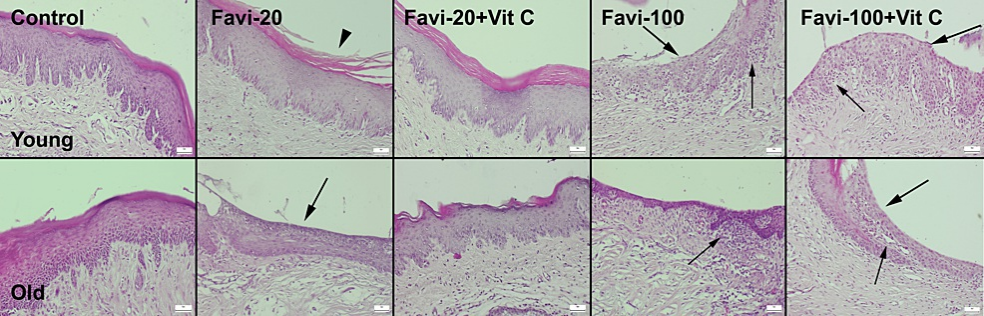
Histopathological appearance of the oral mucosa between the groups. Arrowheads indicate shedding of the keratin layer; thick arrows indicate erosive ulcerative lesions; thin arrows indicate inflammatory cell infiltrations. H&E, scale bars = 50 µm. Favi: favipiravir; Vit C: vitamin C; H&E: hematoxylin and eosin

Normal oral mucosal histology was observed in the young control group. Only a slight increase in the keratin layer was observed in the old control group. No inflammatory reaction was seen in the control group’s oral mucosa. Slight thinning and shedding of the keratin layer were common findings in the young favipiravir 20 mg/kg (Favi-20) group. No erosive or ulcerative lesions were noticed in this group, while slight-to-moderate erosive and (in some areas) ulcerative lesions were observed in the old Favi-20 group. Vit C treatment caused marked amelioration in oral mucosal damage in both young and old Favi-20 + Vit C groups. Healing was more prominent histologically in the young group, but a slight decrease in the thickness of the keratin layer was observable in the old Favi-20 + Vit C group. The most prominent lesions occurred in the favipiravir 100 mg/kg (Favi-100) (young and old) groups. These groups showed erosive and ulcerative lesions in the prominent mouth and gingival mucosa. Although Vit C treatment was effective in these groups, it did not heal the lesions completely. Vit C treatment was found to be more effective in young rats (Figure 2).

Nasal Mucosal Findings

**Figure 3:**
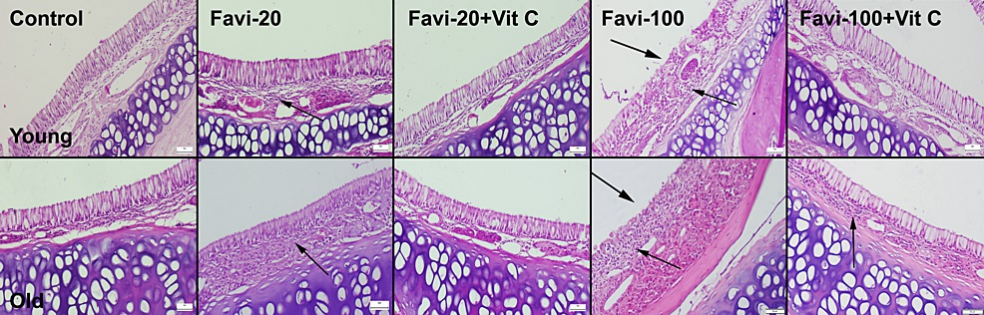
Histopathological appearance of the nasal mucosa between the groups. Thick arrows indicate erosive ulcerative lesions; thin arrows indicate inflammatory cell infiltrations. H&E, scale bars = 50 µm. Favi: favipiravir; Vit C: vitamin C; H&E: hematoxylin and eosin

After the histopathological examination of the nasal mucosa, no pathological findings were seen in the control group, with only a slight increase in mucous-secreted cells in some rats in the old control group. In the Favi-20 groups, a slight-to-moderate inflammatory reaction was observed in both young and old groups. However, the severity of inflammation was more prominent in the old group. Vit C treatment markedly diminished the inflammatory reaction in both young and old Favi-20 + Vit C groups. While Favi-100 application caused mild-to-moderate erosive-ulcerative lesions in young rats, it was observed that the severity of the lesions ranged from moderate to severe in the old rats. Vit C treatment was found to ameliorate lesions almost entirely in the young group, and it caused significant improvements in the old rats (Figure 3).

Immunohistochemical findings

Caspase-3 Immunohistochemical Findings

**Figure 4:**
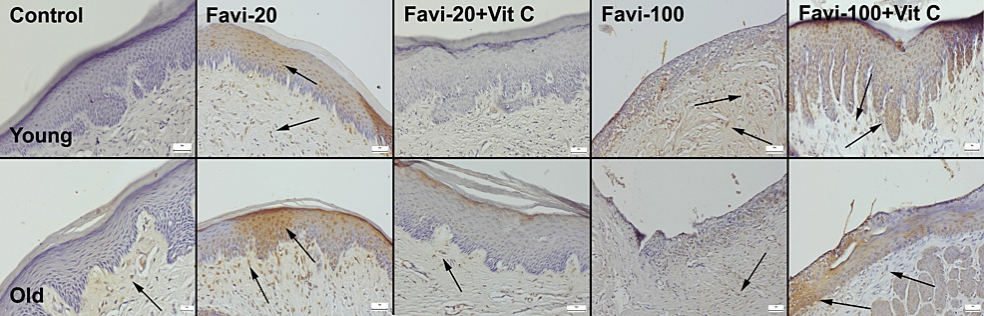
Caspase-3 expressions in oral mucosa between the groups. Arrows indicate positive cells: streptavidin-biotin peroxidase method. Scale bars = 50 µm. Favi: favipiravir; Vit C: vitamin C

Immunohistochemical examination revealed negative expressions in the young control group and slight expressions in the old control group. A moderate increase was seen in expressions in the Favi-20 groups (young and old), and expressions were marked in the old group. Vit C treatment is associated with decreased expressions in both groups. The most marked expressions were observed in the old Favi-100 group and young Favi-100 group. Vit C treatment is associated with a decrease in expression. However, caspase-3 expression was not totally absent (Figure 4).

**Figure 5:**
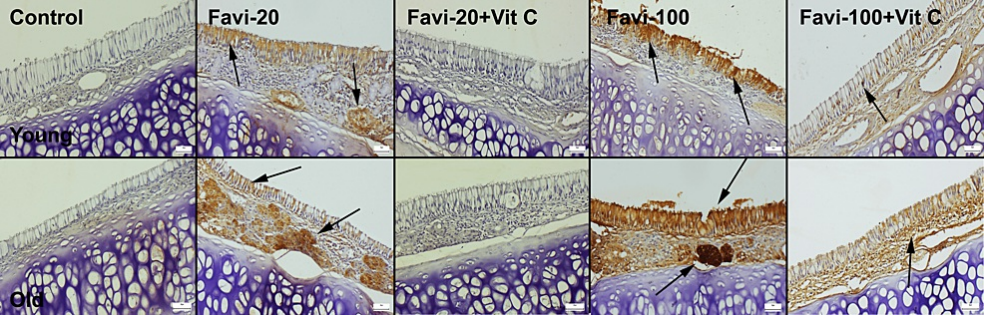
Caspase-3 expressions in the nasal mucosa among the groups. Arrows indicate positive cells: streptavidin-biotin peroxidase method. Scale bars = 50 µm. Favi: favipiravir; Vit C: vitamin C

Similarly, favipiravir administration caused increased caspase-3 expressions, and Vit C treatment decreased the expressions in nasal mucosal cells (Figure 5). An immunopositive reaction was observed in both epithelial and stromal cells.

RANKL Immunohistochemical Findings

**Figure 6:**
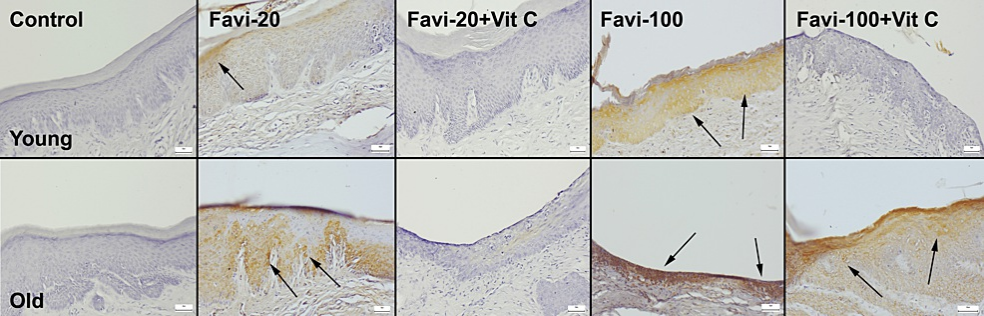
RANKL expressions in the oral mucosa between the groups. Arrows indicate positive cells: streptavidin-biotin peroxidase method. Scale bars = 50 µm. Favi: favipiravir; Vit C: vitamin C

**Figure 7:**
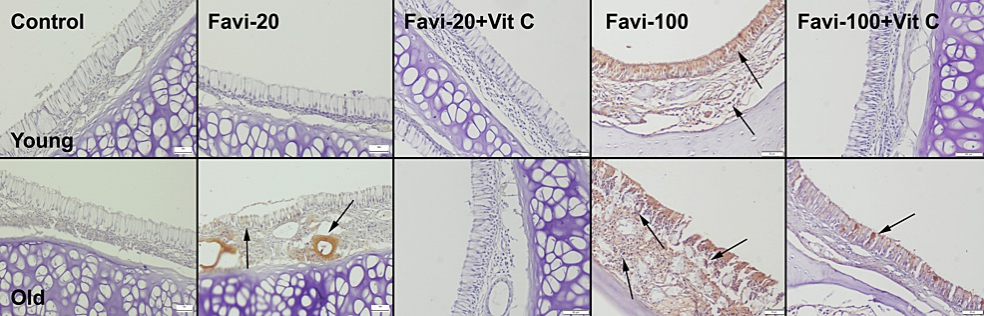
RANKL expressions in the nasal mucosa between the groups. Arrows indicate positive cells: streptavidin-biotin peroxidase method. Scale bars = 50 µm. Favi: favipiravir; Vit C: vitamin C

Immunohistochemistry findings revealed that favipiravir increased the RANKL expression of oral and nasal mucosal cells in young and old rats. Examination indicated that the Favi-20 and Favi-100 groups exhibited increased immunoactivity compared to other groups. The expression of RANKL was more prominent in the Favi-100 group. Treatment of Vit C decreased the RANKL immunoreactivity, with the decrease being especially pronounced in the Favi-20 + Vit C group (Figures 6, 7).

Both epithelial and mesenchymal cells expressed RANKL. The statistical analysis of the immunohistochemical scores is shown in Table 1.

**Table 1:** Statistical analysis results of immunohistochemical scores between the groups. All groups were compared among themselves. However, groups with and without Vit C can be evaluated in the same way among themselves. Favi: favipiravir; Vit C: vitamin C; RANKL: nuclear factor kappa-ligand *: Data are presented as mean ± standard deviation (SD). **: Groups with the same superscript letter on the same column top to bottom are statistically similar, and the differences between groups with different symbols are statistically significant.

	Caspase-3		RANKL		P-value
	Oral mucosa	Nasal mucosa	Oral mucosa	Nasal mucosa	
Control young	0.20 ± 0.13^a^	0.10 ± 0.10^a^	0.20 ± 0.13^a^	0.10 ± 0.10^a^	<0.001
Favi-20 young	1.30 ± 0.48^c^	1.50 ± 0.52^d^	1.50 ± 0.52^c^	1.30 ± 0.48^bc^	<0.001
Favi-20 + Vit C young	040 ± 0.16^ab^	0.80 ± 0.42^bc^	0.80 ± 0.42^b^	0.50 ± 0.16^a^	<0.001
Favi-100 young	2.60 ± 0.51^d^	2.50 ± 0.52^e^	2.70 ± 0.48^d^	2.60 ± 0.51^e^	<0.001
Favi-100 + Vit C young	1.20 ± 0.42^c^	1.50 ± 0.52^d^	1.70 ± 0.48^c^	1.10 ± 0.56^b^	<0.001
Control old	0.50 ± 0.16^ab^	0.40 ± 0.16^ab^	0.40 ± 0.16^ab^	0.30 ± 0.15^a^	<0.001
Favi-20 old	1.40 ± 0.51^c^	1.60 ± 0.51^c^	1.70 ± 0.48^c^	2.10 ± 0.73^d^	<0.001
Favi-20 + Vit C old	0.70 ± 0.48^b^	1.00 ± 0.47^cd^	1.10 ± 0.31^b^	0.60 ± 0.22^a^	<0.001
Favi-100 old	2.80 ± 0.42^d^	2.90 ± 0.31^e^	2.40 ± 0.51^d^	2.90 ± 0.31^e^	<0.001
Favi-100 + Vit C old	1.90 ± 0.56^c^	1.80 ± 0.63^d^	1.60 ± 0.69^c^	2.00 ± 0.66^cd^	<0.001

## Discussion

In this study, the nasal and oral mucosa of old and young rats were pathologically examined and treated for 14 days with favipiravir, a COVID-19 treatment option. Favipiravir had more pronounced side effects on the oral and nasal mucosa in the old rats, and Vit C was less effective in reducing side effects in old rats than in young ones. The COVID-19 outbreak started in China in late December 2019 and spread rapidly worldwide. The pandemic has critically impacted public health systems. In controlling a pandemic for which humanity was unprepared, many drugs approved and marketed for other diseases, including favipiravir, were tried [14]. Favipiravir, a purine nucleic acid analog, is an RNA-dependent RNA polymerase inhibitor and is one of the antiviral medications evaluated after many clinical trials. First produced in Japan, the drug is used experimentally and has been approved for resistant influenza infections [15-17]. The drug has also been effective in treating mild and moderate COVID-19 cases and has been used in many countries [16]. Favipiravir is activated in cells via conversion to its phosphoribosylated form (favipiravir-RTP) and exerts its effect by inhibiting viral RNA polymerase activity [17]. Favipiravir was chosen for this study as it is a drug used to treat COVID-19 in many countries. The effectiveness of the treatment may vary according to the dose, duration of treatment, and cost. Due to its teratogenic effects, favipiravir is not recommended for pregnant women [18]. After obtaining and evaluating the data, it was reported that favipiravir did not significantly benefit treatment and mortality in patients with COVID-19 [14,19]. In addition, several recent studies have reported significant side effects of favipiravir [20-22]. To date, the reported side effects of favipiravir are fever, increased uric acid level, gastrointestinal tract disorders, especially diarrhea, neutropenia, psychiatric symptoms, and enhanced liver function testing [5,20]. In experimental studies, adverse effects of favipiravir on teeth and bone tissue were also observed [7]. This study showed for the first time in an experimental rat model that favipiravir has pathological effects on the oral and nasal mucosa. Pathological findings increased in a dose-dependent manner, especially in elderly rats, and Vit C effectively reduced these effects. The oral mucosal epithelium is a significant barrier that separates the underlying tissues from their external environment. Oral mucosa is a highly functional and specialized mucosa with some differences according to species [23]. Similarly, nasal mucosa has a unique structure, functionally constitutes a physical barrier, and plays an important role in activating the innate and acquired immune system through various cytokines and chemokines. In addition, it is known that cell changes play a role in the pathogenesis of various respiratory system diseases [24]. Caspases that play a significant role in apoptosis have either initiator (caspases-8, 9, and 10) or effector (caspases-3, 6, and 7) functions, depending on their position in the signaling cascade [25,26]. Increased caspase activity has been reported to facilitate pathological processes and negatively affect COVID-19 [27]. This study shows that favipiravir causes increased caspase-3 activity in epithelial and stromal cells in the oral and nasal mucosa. RANKL is a key regulator of osteoclast-induced bone resorption [28]. RANKL plays an important role in osteoclastogenesis and fulfills many functions in the immune system by increasing dendritic cell survival, especially in lymphoid tissue, and regulating lymph node organogenesis [29]. Their expression in tissues indicates an inflammatory reaction, and inhibition of RANKL has been reported to affect amelioration significantly [30]. In this study, RANKL expressions increased in the oral and nasal mucosa of the groups treated with favipiravir, suggesting that the drug increases cell destruction in these cells.

To our knowledge, this study was the first to evaluate the effects of favipiravir on the oral-nasal mucosa in a rat model study. In this study, high-dose favipiravir caused oral-nasal lesions and increased the expression of caspase-3 and RANKL in oral-nasal mucosa cells, and the effect was more pronounced in aged rats. An effective COVID-19 drug is yet to be found, and favipiravir is still among the treatment options. Our results also show that Vit C administration significantly reduces the side effects of favipiravir on the oral and nasal mucosa. The present study suggests that caution should be exercised when using favipiravir, especially in the elderly. If it is to be used absolutely, attention should be paid to lowering the dose and combining it with Vit C.

## Conclusions

In this study, systemic administration of favipiravir at two different doses for 14 days in a rat model produced lesions and increased caspase-3 and RANKL expressions in the oral and nasal mucosa, especially in aged rats, in a dose-dependent manner. The combination of Vit C and favipiravir was more effective in reducing these pathological findings, especially in young rats. More comprehensive studies with different doses and durations are needed to interpret these data for treatment in human patients.
